# Biochemical control after adjuvant radiation therapy for prostate cancer: a unicentric, retrospective analysis

**DOI:** 10.1007/s00066-020-01742-5

**Published:** 2021-01-27

**Authors:** Alexandru-Teodor Zaharie, Matthias Moll, Gregor Goldner

**Affiliations:** Department of Radiation Oncology, Medical University of Vienna, Vienna, Austria

**Keywords:** Prostate cancer, Radical prostatectomy, Adjuvant radiotherapy, Roach formula, Biochemical control

## Abstract

**Purpose:**

To retrospectively evaluate the biochemical no evidence of disease (bNED) and late side effects after adjuvant radiotherapy in prostate cancer patients.

**Methods:**

Patients (*n* = 85) treated with external beam radiotherapy between 1997 and 2013 following radical prostatectomy (RPE) with pathological tumour stage pT2c with positive surgical margins or pT3 and pT4 tumours with or without positive margins who presented with a postoperative and a preradiation prostate-specific antigen (PSA) level below 0.1 ng/ml. The mean dose applied was 66 Gy with conventional fractionation (4 field box-technique). No androgen deprivation therapy was administered, and patients with incomplete data (missing Gleason score, pT stage, or PSA values postoperatively and/or prior to radiation at the presentation at our department) have been excluded from the analysis. Biochemical recurrence was defined as reaching a PSA level > 0.2 ng/ml during follow-up and bNED rates were assessed. In addition, patients were divided into two groups according to the Roach formula for predicting the risk of pelvic node involvement at a cut-off value of 15%. Late urogenital and gastrointestinal side effects (EORTC/RTOG) were assessed.

**Results:**

After a median follow-up of 60 months the bNED rate was 88% at 5 years and 72% at 10 years for all patients. Patients with low risk of lymph node involvement (group < 15%) had a 5 year and 10 year bNED of 97% and 85%, while patients with high risk of positive lymph node involvement (group > 15%) showed corresponding bNED rates of 77% and 52%, respectively. A significant difference according to the Roach stratification was detected (*p* ≤ 0.002). Late urogenital (UG) and gastrointestinal (GI) grade ≥ 2 side effects were detected in 10% and 15%, respectively.

**Conclusion:**

Postoperative radiotherapy with an average dose of 66 Gy to the prostatic fossa following RPE provides excellent tumour control rates with acceptable side effects. Patients with a higher risk of positive lymph nodes (> 15%) according to the Roach formula show significant worse tumour control rates.

## Introduction

Radical prostatectomy and primary radiotherapy are both efficient treatment options for primary localized prostate cancer with a high potential of curation. Nevertheless about 30% of patients develop biochemical recurrence after radical prostatectomy (RPE) [1, 2]. Adjuvant radiation (ART) to the prostatic fossa following radical prostatectomy has shown to provide improved local control and biochemical control for patients with pT3 tumours and or positive surgical margins. This has been proven by four randomised controlled trials [3–9]. However, the bNED (biochemical no evidence of disease) rates within the three randomised trials applying a dose of 60–64 Gy were in range of 70–74% after 5 years. A recently published Finnish trial applied a dose of 66 Gy and reported a bNED rate of 90% after 5 years [6].

The aim of this study was to retrospectively analyse biochemical control after adjuvant radiotherapy applying a comparable high dose as used in the Finnish trial and to confirm these results. In addition a risk stratification using the Roach formula [10] was performed following a previous paper published including more patients with longer follow-up [11].

## Materials and methods

Patients who received adjuvant radiotherapy for localised prostate cancer after radical prostatectomy with no signs of positive lymph nodes or metastasis between 1997 and 2013 in our department were analysed. This includes tumours with pathological tumour stage pT2c with positive surgical margins or pT3 and pT4 tumours with or without positive margins. All of the patients had to have a postoperative and a preradiation prostate-specific antigen (PSA) level below 0.1 ng/ml. No patient received androgen deprivation therapy. Patients have received local radiotherapy limited to the prostatic fossa using a 3D-conformal technique (4-field box). The applied mean total dose was 66 Gy, at 2 Gy single dose, 5 times per week. Dose range was 60–72 Gy, where only 1 patient received a dose of 60 Gy, 2 patients received 72 Gy, and 5 patients 70 Gy. The prostatic region was defined as a volume that includes the surgical limits from the seminal vesicles to the apex, with a security margin to encompass subclinical disease in the periprostatic region [7]. Target volumes were delineated according to the RTOG Consensus from 2010 [10]. A margin of 12 mm in all directions around the clinical target volume (CTV) was added to create the planning target volume (PTV). PSA values, patient history and registration of genitourinary (GU) and gastrointestinal (GI) side-effects using the RTOG/EORTC criteria were assessed every 3 to 6 months in the first 2 years, and once a year thereafter within follow-up. Biochemical recurrence was defined as a PSA level > 0.2 ng/ml during follow-up or the introduction of hormonal treatment after radiation. In addition, patients were stratified by the Roach formula [10]:

$$\mathrm{LN\% }=[(\text{Gleason Score}-6)\cdot 10]+2/3 \mathrm{PSA}$$

for further analysis. One group was defined by patients with a risk stratification for pelvic node involvement < 15% using the Roach formula, whereas the other group is defined as patients with a risk for pelvic node involvement > 15%. All measures of time were calculated from the last day of radiotherapy. The calculation of bNED was performed using the Kaplan–Meier product limit. A *p*-value < 0.05 was considered statistically significant. All of the statistical tests were run in SPSS 26.0. The study was approved by the local ethics committee according to local law regulations (EK Nr. 1698/2019).

## Results

Between 1997 and 2013, a total of 1453 patients received postoperative radiotherapy for prostate cancer at our department. Out of this population, 188 patients were nodal positive or had metastasis (N + or M+) and 390 patients did not have a documented Gleason sore, pT stage, or PSA value (either preoperative, postoperative or prior to radiation); these were also excluded. From the remaining 875 men, 230 have received additional hormonal therapy and 499 had to be excluded due to salvage radiation. Therefore, 146 patients remained, of which 14 had additional pelvic node irradiation and 41 patients received hypofractionated radiation. From the remaining 91 patients, 6 more were excluded for having a PSA value postoperatively or prior to radiation between 0.1–0.2 ng/ml, in order to comply with the current definition of ART, resulting to a cohort of 85 patients.

Most patients had a Gleason score 7 or pT3 stage. Nearly all of the patients included presented with positive surgical margins (95%). A pelvic lymph node dissection was performed in 35% of the patients and the average number of lymph nodes removed was 7. The details of the patient characteristics are shown in Table 1.

**Table 1:** Patients’ characteristics

	Group < 15%	Group > 15%	All patients	ARO 96-02 [3]
*Number of patients*	45	40	**85**	148
*Age at radiotherapy—mean*	63	65	**64**	65
*iPSA—mean*	8.88 ng/ml	9.02 ng/ml	**8.94** **ng/ml**	9.7 ng/ml
*<* *10* *ng/ml*	38 (84%)	13 (32%)	**51 (60%)**	n.a.
*10–20* *ng/ml*	7 (16%)	20 (50%)	**27 (32%)**	n.a.
*>* *20* *ng/ml*	–	7 (18%)	**7 (8%)**	n.a.
***Gleason score***				
$\leq$*6*	28 (62%)	2 (5%)	**30 (35%)**	38%
*7*	17 (38%)	26 (65%)	**43 (51%)**	50%
*8–10*	–	12 (30%)	**12 (14%)**	12%
***Pathological tumour stage***				
*pT2c*	21 (47%)	11 (27.5%)	**32 (38%)**	3%
*pT3*	19 (42%)	26 (65%)	**45 (53%)**	94%
*pT4*	5 (11%)	3 (7.5%)	**8 (9%)**	3%
***Positive resection margins***	42 (93%)	39 (97.5%)	**81 (95%)**	68%
*Lymphadenectomy performed*	13 (29%)	17 (42.5%)	**30 (35%)**	100%
*Numbers of LN removed—mean* *(min-max)*	5 (2–10)	9 (2–44)	**7** **(2–44)**	n.a.
***PSA postoperative ng/ml—mean*** ***(min-max)***	0.02 (0–0.1)	0.02 (0–0.1)	**0.02** **(0–0.1)**	< 0.1 100%
***PSA prior RT ng/ml—mean*** ***(min-max)***	0.02 (0–0.1)	0.03 (0–0.1)	**0.02** **(0–0.1)**	< 0.1 100% < 0.05 80%
*Time RPE to RT in months—median*	3	4	**4**	3.5
*Local Radiation dose Gy—mean* *(min-max)*	66.4 Gy (60–72 Gy)	66.2 Gy (66–72 Gy)	**66.3** **Gy** **(60–72** **Gy)**	60 Gy
*Follow-up in months—median* *(min-max)*	72 (12–174)	54 (1–197)	**60** **(1–197)**	54
*5 year bNED rate*	97%	77%	**88%**	72%

*iPSA* initial prostate specific antigen, *LN* lymph nodes, *RT* radiotherapy, *RPE* radical prostatectomy

**Fig. 1:**
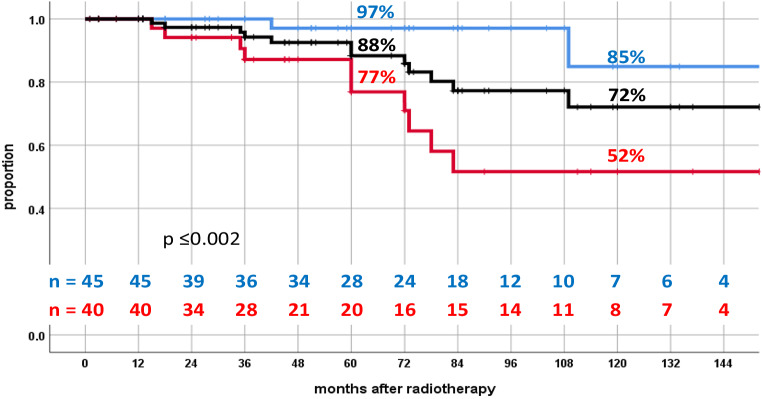
Kaplan–Meier curves showing the bNED value at 5 and 10 years respectively. Group < 15% is represented by the *blue line*, group > 15% is shown through *the red line*, the *black line* represents all patients. *n* number of observations at the corresponding time-points

After a median follow-up of 60 months the bNED rate was 88% at 5 years and 72% at 10 years for all patients. A significant difference according to the Roach stratification could be detected (*p* ≤0.002). Patients with low risk of lymph node involvement (group < 15%) had a 5-year and 10-year bNED of 97% and 85%, while patients with high risk of positive lymph nodes (group > 15%) showed corresponding bNED rates of 77% and 52%, respectively (Fig. 1).

When addressing side-effects, grade 2 or higher toxicity measured by the RTOG/EORTC criteria was registered in 14% of the patients regarding GU effects and 11% when regarding GI effects (Table 2). Grade 3 toxicity was reported in 4 patients: 3 genitourinary and 1 gastrointestinal toxicity. The GI event was a rectal ulcer, which was diagnosed 13 months after radiation and treated successfully conservatively. The GU events were all urethral strictures which developed at different points of time: 2 months, 22 months and 108 months follow-up. One of the patients received a urethral dilation while the other two received a transurethral resection. Postinterventional, the obstructive symptoms relieved in all patients. The urethral dilation had to be repeated after several weeks.

**Table 2:** Late urogenital (UG) and gastrointestinal (GI) side-effects (RTOG/EORTC)

Grade	UG (*n*)	%	GI (*n*)	%
*0*	50	59	53	62
*1*	22	26	22	26
*2*	9	11	8	9
*3*	3	4	1	1
*Missing*	1	1	1	1
*Total*	85	100	85	100

## Discussion

The aim of our study was to evaluate bNED rates after adjuvant radiotherapy for prostate cancer in comparison to published data and to investigate whether the Roach formula can predict the risk of biochemical recurrence. Beside its retrospective nature our study shows additional limitations such as the low number of patients included, the median follow-up is rather short, and the high drop-out rate of the patients during follow-up.

The recently published Finnish randomized trial of adjuvant radiotherapy versus observation after radical prostatectomy included 250 patients in total, 121 in the treatment arm [8]. Hackman et al. reported, after a median follow-up of over 9 years, a 5-year biochemical free survival rate of 90% compared to the 3 randomised clinical trials ARO-096, EORTC 22911 and SWOG 8794 [3–7] reporting about a 5-year biochemical free survival rate of 70–74%. This improvement might be caused by the higher local dose of 66.6 Gy applied in contrast to 60–64 Gy. However, more dose might lead to a better bNED, but this does not necessarily translate into other clinical endpoints like better metastasis-free survival or improved cancers-specific or overall survival. Within our analysis, patients included received a mean dose of 66.3 Gy and we were able to detect a comparable tumour control rate of 88% after 5 years. However, the Finnish trial had several limitations. First of all more than 50% of patients included had a PSA postoperative and before radiotherapy of 0.2–0.5 ng/ml, patients with seminal vesical invasion were excluded and tumour progression was defined by reaching a PSA > 0.4 ng/ml. The patients included in our analysis had a mean PSA after RPE and prior to radiotherapy of 0.02 ng/ml pretty much comparable to the German ARO-96 study (Table 1). In addition we defined biochemical recurrence by reaching a PSA > 0.2 ng/ml. Nearly all our patients (95%) presented with positive surgical margin in contrast to 68% in the German trial. Nevertheless, we were able to confirm the excellent tumour control rates of the Finnish trial validating their results.

We further investigated the impact of the Roach formula on the biochemical free survival by defining two different groups. The two groups were balanced concerning number of patients, age, tumour stage, positive resection margin and postoperative and preradiotherapy PSA (Table 1). All patients received local radiotherapy limited to the prostatic fossa. Patients with low risk of positive lymph nodes according to the Roach formula (< 15%) showed a significant better tumour control rate than patients with high risk of positive lymph node after 5 years (97% vs. 77%; *p* = 0.002). Only 2/45 (4%) patients vs. 10/40 (25%) patients presented with biochemical recurrence. To further improve the results from adjuvant radiotherapy, especially in patients with high risk of lymph node involvement, additional treatment strategies could be evaluated. The RTOG 9413 trial [12] for primary prostate cancer radiotherapy showed superior results for high-risk lymph node positive (Roach > 15%) patients after whole pelvic lymph node radiotherapy. In accordance, one might assume that within postoperative radiotherapy—either adjuvant or salvage setting—such patients might benefit from additional pelvic lymph node irradiation. Spiotto et al. [13] demonstrated such benefit with a 5-year bNED rate of 36% for patients treated at the prostatic fossa only compared to 53% for patients receiving also pelvic lymph node irradiation. Within a multi-institutional retrospective trial from 894 patients Fiorino et al. were able to demonstrate that tumour relapses after postoperative prostate cancer radiotherapy frequently result from clonogens outside the irradiated volume, supporting the choice of lymph-node irradiation, systemic therapy or both for specific subgroups such as Gleason score ≥ 7 patients. Initial results of the RTOG 0534 SPPORT trial were reported at the 2019 ASTRO meeting^.^ This 3 arm trial randomized 1736 patients to salvage radiotherapy to the prostate bed, salvage radiotherapy to the prostate bed and androgen deprivation therapy, or salvage radiotherapy to the prostate bed + ADT + radiotherapy to the pelvic lymph nodes. The 5‑year freedom from progression rate was 71% for salvage radiotherapy to the prostate bed, 81% for salvage radiotherapy to the prostate bed + ADT, and 87% for salvage radiotherapy to the prostate bed + ADT + radiotherapy to the pelvic lymph nodes [13].

Side-effect reporting in our analysis, as well as in the ARO-96 and EORTC trials was done using the RTOG/EORTC grading system. The EORTC study [4] revealed more toxicity in the radiation arm than in the RPE only arm, with an overall grade 3 toxicity of 5.3% versus 2.5%. Grade 2 or higher events occurred with an incidence of 21.3% versus 13.5% regarding the genitourinary tract, and 2.3% versus 1.9% incidence regarding the gastrointestinal tract, reported on a cohort of 512 men. The ARO-96 trial [3] reported one grade 3 genitourinary adverse event and three grade 2 events, while only two grade 2 side-effects regarding the gastrointestinal tract. Overall grade 1 reactions were reported in 21.9% of the 114 men. Our analysis revealed an overall grade 1 toxicity in 34% of the patients, with an increased incidence of overall grade 2 adverse events in around 19% and grade 3 adverse events in 5% of the men. This increase compared to the German trial might be due to the higher applied dose.

## Conclusion

We were able to confirm the results from the Finnish trial showing improved biochemical recurrence free survival rates for patients receiving adjuvant radiotherapy after radical prostatectomy with a local dose of at least 66 Gy with an acceptable toxicity profile. Furthermore, patients with higher risk of positive lymph nodes (> 15%) according to the Roach formula show significant worse tumour control rates.
